# Beyond Opioids: A Review of Suzetrigine for Acute Pain Management

**DOI:** 10.3390/ijms26209865

**Published:** 2025-10-10

**Authors:** Andrew Pham, Hua Yep, Sebastian Wozniak, Anisha Javvaji, Eman Nada, Sergio Bergese

**Affiliations:** 1 Department of Anesthesiology, Stony Brook Hospital, Stony Brook, NY 11794, USA; andrew.pham@stonybrookmedicine.edu (A.P.); hua.yep@stonybrookmedicine.edu (H.Y.); eman.nada@stonybrookmedicine.edu (E.N.); 2 Renaissance School of Medicine, Stony Brook, NY 11794, USA; sebastian.wozniak@stonybrookmedicine.edu (S.W.); anisha.javvaji@stonybrookmedicine.edu (A.J.)

**Keywords:** suzetrigine, Nav1.8, nonopioid, acute pain, sodium channel

## Abstract

The ongoing opioid epidemic in the United States highlights the need for novel analgesics with reduced risk of misuse, dependence, and adverse central nervous system effects. Suzetrigine (trade name Journavx), a first-in-class selective Nav1.8 (a sodium channel expressed in peripheral nociceptors) inhibitor, was approved by the FDA in early 2025 and marketed as the first non-opioid analgesic in over two decades. Current studies have shown that suzetrigine has potential in treating acute pain perioperatively for minimally to moderately painful ambulatory procedures. However, suzetrigine appears less potent than hydrocodone-acetaminophen in this context, and it remains unclear how effective suzetrigine is in treating more severe postoperative pain. Notably, all current studies are limited to short durations of treatment; further studies will be required to delineate suzetrigine’s long-term efficacy, safety, and addiction potential to be utilized in the management of chronic pain patients. This article provides a review of the current literature available on the use of suzetrigine in treating acute pain.

## 1. Introduction

With the designation of pain as the fifth vital sign in the mid-1990s, conservative pain management was replaced with more aggressive, largely opioid treatment in the United States [1]. Multiple factors contributed to this transition, including aggressive marketing of opioid medications such as OxyContin and campaigns minimizing the perceived risk of addiction [2]. By the end of the 1990s, prescription opioid misuse in the setting of poor outpatient surveillance and prescription management had become the leading driver of opioid overdose deaths [3]. In the early 2000s, patient-reported pain scores were incorporated into hospital quality metrics through post-care questionnaires such as the Hospital Consumer Assessment of Healthcare Providers and Systems (HCAHPS) survey, which tied greater patient satisfaction, and by extension pain control, to increased reimbursement through Medicare, which encouraged liberal opioid prescription [4]. While deaths due to overdose in the United States have, for the first time, declined in the past year due in part to changes in legal policy and prescriber strategy, the annual overdose death toll, a majority of which is opioid-related, is still projected to exceed 70,000 by the Center for Disease Control [5]. While the opioid epidemic was largely related to mismanagement and disinformation, opioids have been villainized for better or worse. With public opinion shifting against opioids, pharmaceutical companies have sought to develop new nonopioid, potentially nonaddictive, analgesics.

One such medication, suzetrigine (trade name Journavx, also referred to as VX-548), was recently FDA-approved at the beginning of 2025 [6]. It is the first non-opioid analgesic approved in over two decades and shows promise in the treatment of moderate to severe acute pain without central side effects such as sedation, respiratory depression, or addiction after short-term use [7]. It should be noted that no studies assessing long-term use in chronic pain patients have been performed to date. Suzetrigine exerts its effect as a selective inhibitor of Nav1.8, a voltage-gated sodium channel found in peripheral nociceptors, implicated in pain signaling [8]. In this article, we intend to outline the mechanism and pharmacology of suzetrigine as well as review the current studies regarding its use in the treatment of acute pain.

## 2. Mechanism of Action

Pain is not a discrete event but rather the product of a multistage process encompassing transduction, transmission, and perception. This process of detecting painful stimuli is called nociception, and pain refers to the actual experience perceived by a person as a result of the sensory and emotional components. Potentially harmful stimuli such as heat, mechanical injury, or inflammation are detected by peripheral nociceptors and converted into electrical signals in transduction. These impulses travel through small-diameter Aδ and C fibers to the dorsal horn of the spinal cord, where excitatory neurotransmitters, including glutamate, substance P, and calcitonin gene-related peptide (CGRP), activate second-order neurons [9]. Ascending pathways, such as the spinothalamic tract, travel to the ventral posterior nuclei in the thalamus. From the thalamus, the signal is relayed to the somatosensory cortex, where location and intensity are perceived. These signals are also relayed to various parts of the brain that influence the experience of pain. Projections to the limbic system are responsible for the emotional component involved with pain, while inputs to the prefrontal cortex affect attention and behavioral response [10]. At each stage, neuronal excitability is controlled by ion channel function, particularly voltage-gated sodium channels. These enable the initiation and propagation of action potentials.

Voltage-gated sodium channels (Navs) initiate and propagate action potentials in neurons. Among the nine subtypes (Nav1.1–Nav1.9), Nav1.7, Nav1.8, and Nav1.9 are found in peripheral sensory neurons [8]. Human genetic studies have highlighted the importance of these channels: gain-of-function mutations in SCN9A (Nav1.7) cause erythromelalgia (severe burning pain), while loss-of-function mutations cause congenital insensitivity to pain [11]. Nav1.8, encoded by SCN10A, mediates persistent sodium current in C-fibers, is critical in sustaining high-frequency firing, and is involved in nociception [12].

**Figure 1:**
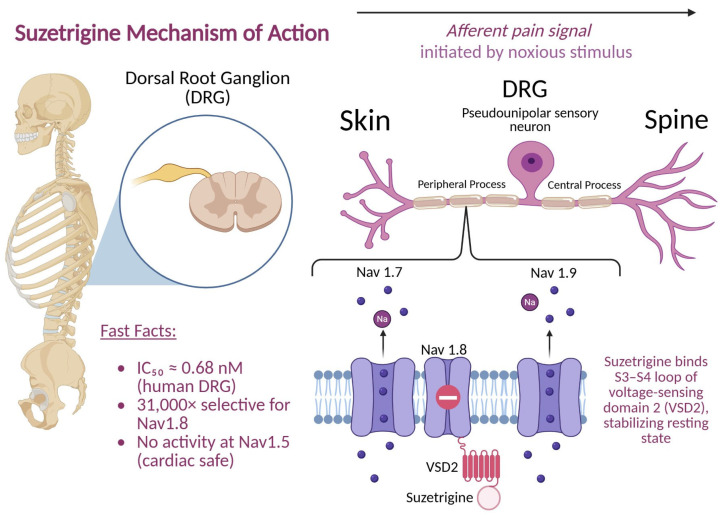
Mechanism of action of suzetrigine. Suzetrigine selectively inhibits the Nav1.8 peripheral sodium channel.

Nav1.8 channels are found in the dorsal root ganglion (DRG) and are involved in mediating acute pain [13], Figure 1. The absence of Nav1.8 has been shown to dramatically lower acute pain responses in animal models [13]. Because Nav1.8 channels are not present in the CNS, the central side effect risk is theoretically minimal. Vertex Pharmaceuticals utilized this information to develop suzetrigine as a highly selective Nav1.8 antagonist in humans [7].

Suzetrigine inhibited human Nav1.8 currents with sub-nanomolar potency (IC_50_ ≈ 0.68 nM in human DRG; ~0.75 nM in monkey), while much less potent in rodents. Moreover, it exhibits over 31,000-fold selectivity for Nav1.8 versus other Nav subtypes and 180+ molecular targets. This selectivity minimizes off-target effects, especially on cardiac (Nav1.5) muscle. Using chimeric Nav1.8/Nav1.2 constructs, the binding site was mapped to the voltage-sensing domain 2 (VSD2)—particularly the S3–S4 loop—highlighting a unique binding motif [7]. Binding assays showed a dissociation constant (Kd) ≈ 65 nM for purified VSD2, indicating direct interaction. Electrophysiological assays revealed suzetrigine preferentially binds Nav1.8 in the “closed” resting state, stabilizing it and producing tonic inhibition. A 5 Hz train produced consistent inhibition across pulses, without use-dependence accumulation; this aligns with an allosteric mechanism rather than pore occlusion. Variants like LTGO-33 and A-887826 show differential state dependence; suzetrigine uniquely stabilizes the closed state blockade.

VX-548 exhibits strong state-dependent inhibition—especially in the closed/inactivated states [13,14]. This research also demonstrated that suzetrigine has reverse use-dependence, meaning its inhibition of Nav1.8 could be relieved by repetitive depolarizations. However, suzetrigine inhibition relief required large depolarizations with a time constant of ~40 ms. Reinhibition at negative voltages occurred at a rate that was almost proportional to drug concentration. Relief of inhibition reflected dissociation of the drug from the channel, and reinhibition reflected rebinding. This means that suzetrigine has a strong state dependence with weak binding to channels with fully activated voltage sensors and very tight binding to channels with voltage sensors in the resting state, which is different from other sodium channel inhibitors. Combined, these studies validate that suzetrigine effectively reduces nociceptor excitability via selective Nav1.8 blockade.

Suzetrigine is orally active, dosed twice daily, with a peak plasma concentration of about 3 h and a steady state of about 3 days [15]. It is almost 99% protein-bound, with a large volume of distribution, metabolized mainly by CYP3A4, and contraindicated with strong CYP3A inhibitors—likely due to increased exposure [16]. It also induces CYP3A, posing potential drug–drug interactions. Further evaluation of suzetrgine’s active metabolites and their clinical significance is necessary. Bertoch et al. reported two Phase 3 randomized controlled trials—bunionectomy and abdominoplasty—comparing suzetrigine with placebo and hydrocodone/acetaminophen [17]. Suzetrigine demonstrated opioid-comparable analgesia, significantly reducing pain scores and increasing proportions of patients with ≥30% pain relief, outperforming placebo with fewer side effects. A third single-arm Phase 3 study showed robust analgesia across surgical and non-surgical acute pain settings, reinforcing broad applicability [18]. Adverse events were mild and limited to peripheral manifestations: itch, muscle spasms, elevated creatine kinase, rash, nausea, and headache. Importantly, with more than 2400 participants, no signs of CNS-related impairment, respiratory depression, or addictive behaviors were observed—even during 30-day administration followed by abrupt withdrawal in animal studies. Suzetrigine exemplifies a mechanism-driven analgesic: a potent, allosteric inhibitor of peripheral Nav1.8, stopping nociceptor firing with sub-nanomolar potency and unprecedented selectivity [7].

## 3. Pharmacology

The chemical name for suzetrigine is 4-[[(2R,3S,4S,5R)-3-(3,4-difluoro-2-methoxyphenyl)-4,5-dimethyl-5-(trifluoromethyl)oxolane-2-carbonyl]amino]pyridine-2-carboxamide. The molecular formula is C21H20F5N3O4, and it has a molecular weight of 473.4 g/mol. The molecular structure consists of a tetrahydrofuran (THF) core substituted at five positions, contributing to its unique chemical and pharmacological profile [19].

Suzetrigine is formulated as an immediate-release, film-coated oral tablet under the brand name Journavx. Each tablet contains 50 mg of active drug [20]. No alternative formulations are currently approved by the FDA. Suzetrigine dosing for acute pain was established through two Phase 2 trials in bunionectomy and abdominoplasty, which tested various combinations of loading dose + maintenance dose every 12 h for 36 h. Only the highest dosing regimen tested (100 mg loading followed by 50 mg every 12 h) showed a statistically significant reduction in pain versus placebo [21]. Thus, this dosing was selected for phase 3 trials.

Nonclinical studies showed that suzetrigine exhibits significant species-dependent potency at Nav1.8. In vitro assays in DRG neurons showed an IC_50_ of 0.68 nM in humans and 0.75 nM in monkeys, whereas rodent Nav1.8 channels were approximately 80-fold less sensitive, with an IC_50_ of 56 nM7. Because of this reduced potency, traditional rodent pain models were not predictive of suzetrigine’s clinical activity, and nonhuman primate and human data were prioritized for translational assessment. The major human metabolite, M6-SUZ, also retains potent activity at Nav1.8 (IC_50_ ~2.5 nM in human DRG neurons) [22]. This indicates the likely contribution of both the parent drug and the metabolite to overall pharmacologic activity. This species selectivity highlights the necessity of human-specific data in the development of peripherally acting sodium channel inhibitors.

**Figure 2:**
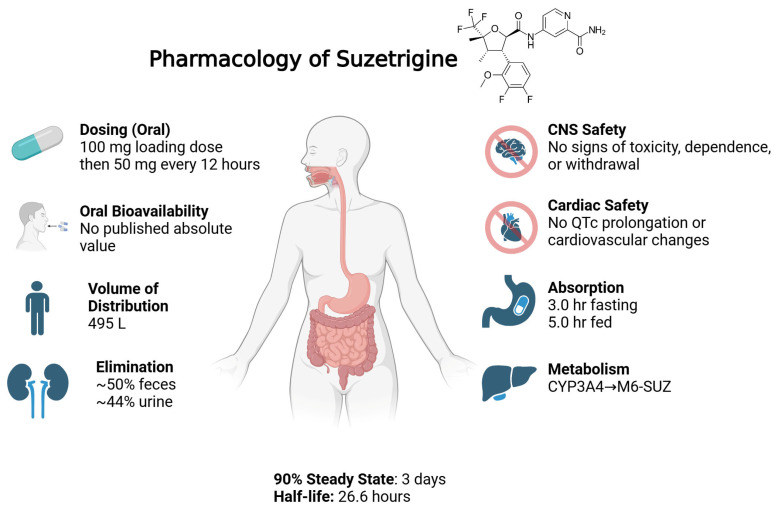
Pharmacology of suzetrigine. Pharmacologic properties at a glance.

As per the FDA drug label, following oral administration, the median time to peak plasma concentration (Tmax) is 3.0 h under fasting conditions and delayed to 5.0 h when taken with food. Steady-state levels are reached within 3 days [23]. Preclinical studies demonstrated oral bioavailability of 69.5% in monkeys and 68.1% in dogs; however, absolute bioavailability in humans has not been disclosed [22]. The drug exhibits extensive protein binding of greater than 99% (to both albumin and alpha-1 acid glycoprotein), which suggests limited free circulation and likely contributes to a prolonged effective half-life of 23.6 h [22]. It has a large volume of distribution of 495 L, which shows substantial tissue penetration. The mean plasma clearance is 13.9 L/h. As per the FDA integrated review, the body weights of patients tested (44–126 kg) and ages (18–75 years) did not have any clinically meaningful effect on the pharmacokinetics of suzetrigine [22], Figure 2.

Preclinical studies in animal models show that suzetrigine does distribute to the central nervous system in both monkeys and dogs [22]. However, studies in rats and monkeys demonstrated no signs of CNS toxicity, abuse potential, or physical dependence with suzetrigine, even at exposures exceeding 50 times the human therapeutic levels [7]. In repeat-dose toxicity and withdrawal studies, suzetrigine did not induce neurobehavioral changes, withdrawal symptoms, or alterations in body weight, temperature, or motor activity, in contrast to morphine-treated controls [7]. This highlights the drug’s high specificity for the peripherally located NaV1.8 channels. Consistent with these findings, Phase 3 trials showed a low and comparable incidence of adverse events associated with abuse liability between suzetrigine and placebo [7]. No meaningful misuse, dependence, or withdrawal was detected across the trials [7].

Cardiovascular safety was also confirmed in monkeys monitored via telemetry. No changes in blood pressure, ECG parameters, or respiratory function were observed after both single and repeated dosing [7]. A phase 1 study was conducted to determine suzetrigine’s effect on the QTc interval [24]. According to the drug label, there was no clinically significant QTc prolongation at up to two times the maximum recommended dose [20].

Suzetrigine undergoes hepatic metabolism primarily by the cytochrome P450 3A4 (CYP3A4) system, resulting in oxidative transformation and the formation of its main active metabolite, pyridine N-oxide (M6-SUZ) [22]. This metabolite is approximately 3.7-fold less potent than the parent compound and is further metabolized by CYP3A4 [22]. Given this pathway, caution is warranted when suzetrigine is administered with other drugs that are substrates, inducers, or inhibitors of CYP3A4. Common analgesic CYP34A substrates include acetaminophen and codeine, potentially complicating multimodal use. Suzetrigine is contraindicated with strong CYP3A4 inhibitors such as ketoconazole, as suzetrigine plasma levels may increase substantially, potentially leading to toxicity [20]. Suzetrigine itself is a moderate inducer of CYP3A4, which may lead to reduced plasma levels of concurrently administered CYP3A4 substrates [20].

The interactions between suzetrigine and transporters appear to be limited. In vitro studies showed that suzetrigine itself is not a substrate for transporters such as P-glycoprotein (P-gp), breast cancer resistance protein (BCRP) or the hepatic uptake transporters OATP1B1 and OATP1B322. Although M6-SUZ was shown to be a P-gp substrate, it does not appear to be clinically significant. This confirmation was provided through an open-label clinical study demonstrating that suzetrigine does not significantly alter the pharmacokinetics of digoxin, a sensitive P-gp substrate [22]. This finding also provides reassurance that P-gp substrates can be safely co-administered without the need for dose adjustment. Both the parent drug and M6-SUZ showed inhibitory activity against OATP1B1, OATP1B3, and OAT3 in vitro, but these effects were weak and considered unlikely to translate into clinically meaningful interactions at therapeutic concentrations.

Elimination of suzetrigine and its metabolites occurs through both hepatic and renal pathways. Based on mass balance studies in animals and phase 1 data, approximately 50% of the administered dose is recovered in feces and 44% in urine, mostly as metabolites. The excretion of the unchanged parent drug is negligible [22]. Although clinical trials did not show any significant elevations in liver enzymes, those with moderate hepatic disease (Child-Pugh-B) may require a reduction in dose [22]. Peak concentrations increased by 31% in clinical trials [22]. Those with severe hepatic disease (Child-Pugh-C) are contraindicated, as they were not studied during clinical trials [23]. Similarly, the drug’s use in patients with severe renal impairment (eGFR < 15 mL/min) is unstudied as well [23]. In a phase 2 study for diabetic peripheral neuropathy, suzetrigine was associated with dose-dependent increases in serum creatinine. However, in acute pain trials, changes in renal markers were transient and clinically insignificant [22]. Additional studies in populations with baseline renal impairment are warranted, especially in the setting of long-term use.

Results of preclinical studies of reproduction and development varied by sex. While fertility in male rats was unaffected at doses up to 1000 mg/kg, female rats showed increased preimplantation and postimplantation loss at doses as low as 15 mg/kg, with reduced fetal weights also noted. Preimplantation loss was found to be reversible after a washout period. In rats, prenatal exposure led to shortened gestation, increased pup mortality, and lower birth weights at clinically relevant exposure levels [22]. To date, no clinical studies have evaluated the effects of suzetrigine on pregnancy or fertility in humans.

## 4. Studies Regarding the Use of Suzetrigine for Analgesia

Previous studies concerning suzetrigine consist primarily of clinical trials funded by industry due to the relatively recent FDA approval of the pharmaceutical.

In a pair of double-blinded, randomized, controlled phase 2 trials, Jones et al. investigated the efficacy and safety of suzetrigine in patients receiving bunionectomy or abdominoplasty procedures. Abdominoplasty patients were randomly assigned to high-dose suzetrigine (100 mg loading dose with 50 mg redosing every 12 h), medium-dose suzetrigine (60 mg loading dose with 30 mg redosing every 12 h), hydrocodone–acetaminophen (5–325 mg every 6 h), or placebo groups, while bunionectomy patients were randomly assigned to the aforementioned groups as well as a low-dose suzetrigine group (20 mg loading dose with 10 mg redosing every 12 h) and received treatment for 48 h. The primary outcome measure was pain-intensity difference over a period of 48 h (SPID48), a numeric value calculated by the authors through the weighing and summing of 19 numeric pain rating scale (NPRS) scores obtained at predetermined times in the perioperative period. SPID48 for patients receiving high-dose suzetrigine was increased versus the placebo group, indicating improved analgesia. Notably, no statistically significant differences were found between the groups receiving medium- and low-dose suzetrigine versus the placebo. Secondary outcome measures efficacy was investigated with the same SPID metric over 24 h (SPID24), but no statistically significant differences for any group versus placebo were found. Safety was measured through the incidence of adverse events. In this study, suzetrigine was associated with increased risk of headache and constipation but not respiratory depression or altered mental status compared to placebo. The authors concluded that oral suzetrigine was able to provide improved analgesia after bunionectomy and abdominoplasty up to 48 h post-procedure, but was associated with the aforementioned minor side effects [21].

Bertoch et al. performed a similar study to Jones et al. as a phase 3 trial again investigating the use of suzetrigine to treat acute pain after abdominoplasty or bunionectomy. Patients receiving either procedure were randomized into a suzetrigine (100 mg loading dose with 50 mg redosing every 12 h), hydrocodone-acetaminophen (5–325 mg every 6 h), or placebo group. Treatment was given up to 48 h. Notably, bunionectomy patients received popliteal blocks in all groups. In contrast, patients in the abdominoplasty groups did not receive any regional nerve blocks. The primary outcome measure was again SPID48 between suzetrigine treatment groups and placebo control groups, while secondary outcome measures were SPID48 in suzetrigine groups against the hydrocodone-acetaminophen groups, as well as time until there was a 2-point decrease in NPRS scores in suzetrigine treatment groups versus that of the placebo group. The suzetrigine treatment group showed an increased SPID48, that is to say, a higher differential in pain, on average, as compared to the placebo group in both bunionectomy and abdominoplasty arms, while there were no statistically significant differences in SPID48 between the suzetrigine and hydrocodone-acetaminophen groups. The suzetrigine group also displayed a decreased time to 2-point reduction in NPRS score versus the placebo group in both procedure groups. The authors concluded that suzetrigine was able to mitigate pain after bunionectomy and abdominoplasty in the 48 h post-procedure period and that this pain reduction was similar to that of low-dose hydrocodone-acetaminophen of 5–325 mg every 6 h [17].

McCoun et al. performed a phase 3, multicenter, single-arm study to ascertain whether suzetrigine was safe and effective in treating pain, both surgical and nonsurgical. Patients with moderate to severe pain, as well as 4 or greater on the NPRS, received high-dose suzetrigine at the same dose as in previously outlined studies for up to 14 days. Surgical patients were eligible for the study for ambulatory procedures of varying specialties if pain was expected to subside within 3 days, and if admission was required, it would be for less than 1 day. Nonsurgical outpatients were required to have new, undiagnosed acute pain when evaluated in the outpatient setting. Exclusion criteria were notable for American Society of Anesthesiologists (ASA) classification 3 or greater, sleep apnea, peri-pregnancy, concurrent analgesic use that cannot be discontinued for at least five half-lives or two days, and confounding painful physical conditions. The primary outcome measure was safety through adverse events and consisted primarily of minor (27.7%) to moderate (8.2%) events such as headache and constipation. The secondary outcome measure was a qualitative patient-reported self-assessment of the effectiveness of suzetrigine for pain control. Patients responded poor, fair, good, very good, or excellent. A total of 83.2% of patients in the study felt that suzetrigine provided good or better pain relief. Notably, 91.2% of nonsurgical patients responded good or better versus 82% of surgical patients in the study. The authors concluded that suzetrigine was both safe and effective for analgesia of acute moderate to severe pain [18].

## 5. Discussion and Clinical Applications

Suzetrigine is currently FDA-approved for the treatment of moderate-to-severe acute pain [22]. Current studies have shown suzetrigine’s efficacy in treating pain after ambulatory bunionectomy and abdominoplasty, which are generally considered minimally to moderately painful procedures [17,21]. Based on the current literature, it is unclear how effective suzetrigine will be in treating pain for more notoriously painful procedures such as shoulder or spine surgery. Notably, Bertoch et al. found that suzetrigine at a high dose (100 mg loading dose with 50 mg redosing every 12 h) provided similar analgesia to hydrocodone-acetaminophen at a low dose (5–325 mg every 6 h), which implies that suzetrigine is less potent. It is not uncommon for postoperative patients in post-anesthesia care units (PACU) to require multiple doses of narcotics in a short time interval for severe hyperacute pain. Given that suzetegrine was found to be effective only at the high dose, it is likely that suzetegrine cannot be used to the same effect as opioids in this context while maintaining a favorable side effect profile. Further studies would be helpful in ascertaining whether suzetrigine can be safely given at even higher doses to improve analgesia.

McCoun et al. assessed suzetrigine in a broader acute pain context, both surgical and non-surgical, expanding upon previous studies. However, this study had notable limitations, including the lack of placebo control, elective and thus sporadic over-the-counter analgesic adjuvant use, and a high proportion of ambulatory surgery patients versus outpatients, limiting generalizability to non-surgical pain [18]. In addition, patients in the McCoun et al. study self-reported qualitative pain assessments, which, combined with the above limitations, weaken any conclusions that can be drawn from the study.

All previous studies performed evaluated the use of suzetrigine in a relatively healthy (mostly if not all ASA class 1–2), overwhelmingly female patient population due to the demographic of patients who receive elective bunionectomy and abdominoplasty procedures [17,18,21]. While suzetrigine is likely similarly efficacious in both men and women, there have been previous studies that noted differences in responses to pharmaceutical analgesia between the sexes, which varied from medication to medication [25]. Additional studies with a more balanced patient sex distribution are warranted to elucidate if suzetrigine analgesia differs between men and women.

Bertoch et al. utilized popliteal blocks for the bunionectomy arms of the study, which obfuscates the degree of suzetrigine’s analgesic effects. After a popliteal block, patients are expected to be reasonably numb and should not feel intolerable discomfort for the duration of the block, especially for a minimally to moderately painful procedure such as a bunionectomy. While there was a statistically significant difference between the suzetrigine treatment and placebo control group, it is uncertain whether this effect is synergistic with the block or purely additive. The choice of popliteal block over ankle block is also interesting, as these patients were ambulatory, and there is more motor blockade with the popliteal block. Ankle block patients would be expected to be able to mobilize earlier, which would be favorable in this surgical setting. Of note, popliteal blocks were found to be more analgesically effective and opioid sparing for bunionectomy in some studies [26].

As previously mentioned, all current studies have evaluated suzetrigine in the acute postoperative pain setting. As a result, patients received suzetrigine for only short durations at a set dose. Because of the relatively short treatment time, addiction would be unlikely to occur in these patients. As opioid addiction is more commonly seen in the chronic pain setting with long-term, high-dose use, further studies evaluating suzetrigine under these parameters would be necessary to compare and assess suzetrigine’s addiction potential. Furthermore, it is well known that acute pain and chronic pain are discrete entities with separate pathophysiology [27]. While previous studies have shown suzetrigine’s efficacy in the acute pain setting, it is unclear whether this drug will be similarly effective in treating chronic pain due to these pathophysiologic differences.

Suzetrigine’s twice-daily oral dosing, minimal CNS effects, and lack of respiratory depression make it suitable for both outpatient recovery and early mobilization protocols, which may be particularly attractive in an ambulatory surgery center to improve PACU turnover and reduce the incidence of at-home respiratory complications. It may still prove useful in an inpatient setting as part of a multimodal regimen, but further studies would be needed to assess suzetrigine’s role and efficacy. The lack of an IV formulation prevents its use in the immediate postoperative period and in patients who must remain nil per os, such as those receiving intra-abdominal surgery or those requiring continued sedation after surgery. According to the manufacturer, suzetrigine may not be crushed, so patients who cannot swallow or require any form of gastric tube are unable to receive the medication [22].

Suzetrigine has the potential to act as a primary non-opioid agent alongside regional anesthesia, nonsteroidal anti-inflammatory drugs (NSAIDs), acetaminophen, and muscle relaxants in multimodal regimens; however, further study is required to investigate the cost-risk-benefit of such an addition. Notably, there is limited data for the use of suzetrigine in patients with severe hepatic and renal impairment, which raises concerns for use in these populations currently. As suzetrigine is a CY3P4A substrate and inducer, careful consideration of drug interactions with other medications that modulate or are metabolized by CY3P4A, such as antibiotics and direct oral anticoagulants, is required when administering suzetrigine in complex patients [28].

Overall, suzetrigine appears to be clinically useful in treating short-lived moderate postoperative pain in a limited subset of healthy patients to avoid the unwanted central side effects of opioids. Based on the current literature, it is difficult to assess whether the pain control provided by suzetrigine is comparable to that of opioids, but it seems unlikely to replace opioids in patients with severe pain who may require high opioid doses for analgesia.

## 6. Conclusions

Suzetrigine is a promising, novel non-opioid analgesic that exerts its effect by specifically inhibiting Nav1.8, a peripheral sodium channel implicated in pain signaling. Because it is not active at cardiac and central sodium channels, it does not exhibit the well-established cardiotoxic and neurotoxic effects of local anesthetics. Additionally, suzetrigine use was not associated with central side effects such as respiratory depression or sedation. Studies have demonstrated that suzetrigine is both safe and effective for the treatment of acute pain, albeit in relatively healthy, ideal patients undergoing a limited breadth of procedures. The current literature available consists of relatively limited studies with patients experiencing minimal to moderate levels of pain, mostly post-surgically. Because these studies had short durations of treatment at a static dose, suzetrigine’s efficacy, safety, and potential for dependence at escalating doses and in longitudinal pain management are unclear and require investigation. Further studies are required to elucidate whether suzetrigine is an effective adjunct in a multimodal pain regimen with and without opioids, as well as in the treatment of severe pain following more complex, painful procedures such as spine surgery or in outpatients with underlying chronic conditions such as complex regional pain syndrome. Notable ongoing studies are exploring suzetrigine’s role in chronic pain conditions such as diabetic peripheral neuropathy and lumbar radiculopathy, as well as a Phase 4 trial focused on reconstructive and aesthetic surgeries [29,30,31]. Data from these studies are not currently available as they are not complete, but each represents a potential source for answers to previously discussed questions about suzetrigine’s long-term sequelae and role in more complex and severe pain management in a variety of conditions and procedures. Suzetrigine has been shown to be effective in a limited niche and must be tested further to expand its role in the greater context of pain management.

## Data Availability

No new data were created or analyzed in this study. Data sharing is not applicable to this article.

## Abbreviations

The following abbreviations are used in this manuscript:

HCAHPS	Hospital Consumer Assessment of Healthcare Providers and Systems
FDA	Federal Drug Administration
Nav	voltage-gated sodium channel
CNS	central nervous system
DRG	dorsal root ganglion
VSD2	voltage-sensing domain 2
CYP3A4	cytochrome P450 3A4
M6-SUZ	pyridine N-oxide
RCT	randomized controlled trial
THF	tetrahydrofuran
ECG	electrocardiogram
NPRS	numeric pain rating scale
SPID	pain-intensity difference
SPID24	pain-intensity difference over a period of 24 h
SPID48	pain-intensity difference over a period of 48 h
ASA	American Society of Anesthesiologists
NSAID	nonsteroidal anti-inflammatory drugs
PACU	Post-anesthesia care unit
